# Association of Academic Performance With Obesity and Unhealthy Lifestyle Among Female University Students

**DOI:** 10.7759/cureus.21561

**Published:** 2022-01-24

**Authors:** Ala M Aljehani, Shaima A Banjar, Ghada A Alshehri, Taif F Alnojaidi, Shahad M Alkhammash, Farah A Almomen, Omar K Alolayan, Ghassan A Bagazi, Abdulrahman S Bamhair

**Affiliations:** 1 College of Medicine, Al-Imam Mohammad Ibn Saud Islamic University, Riyadh, SAU; 2 Family and Community Medicine, Al-Imam Mohammad Ibn Saud Islamic University, Riyadh, SAU

**Keywords:** body mass index, unhealthy habits, obesity, grade point, physical activity, academic performance

## Abstract

Aim

This study aims to examine the relationship between obesity and academic performance and to investigate the relevance between an unhealthy lifestyle and academic performance, which may exist among obese and non-obese female students in the College of Science and College of Medicine of Imam Mohammad Ibn Saud Islamic University.

Method

The study is observational, descriptive, and cross-sectional in nature and covers a sample of 328 female students aged 19 to 23 years. They were randomly selected from the College of Science and College of Medicine. Sample collection was conducted from December 12, 2020 to June 15, 2021.

Results

The result indicates that obesity did not influence academic performance. The association between obesity and academic performance was non-significant (*p* = 0.335). In the College of Science, grades in physics and math were analyzed, which reflected a weak association with obesity (*p *= 0.893 and *p *= 0.872, respectively). Various factors exerted a positive impact on academic performance, such as eating yogurt at least 1-5 times per week (*p* < 0.05) and spending less than 4 hours on social media or watching TV (*p* < 0.05).

Conclusion

Academic performance is influenced by many factors. Eating yogurt more frequently and watching TV and less time for social media are the factors with the largest influence on academic performance. However, obesity does not influence academic performance. In summary, physical activity and a healthy diet exerted no direct effect on academic performance.

## Introduction

Obesity is considered one of the most widely-occurring conditions, where the rates of obesity have become a health concern for governments worldwide. Scholars found obesity to be a highly-related to other non-communicable diseases [1]. Obesity also impacts on psychosocial status. Individuals who suffer from obesity struggle with issues related to their mood, self-esteem, quality of life, and body image [2]. In Saudi Arabia, the percentages of obesity are vigorously rising. Althumiri et al. [3] conducted a study in 2020 and estimated a BMI of 30 kg/m^2^ or more than 24.7%, where females displayed a higher prevalence than males (25.5% and 17.9%, respectively). A positive relationship was found between healthy body weight and improved academic performance [4]. Many studies proved the impact of unhealthy lifestyle and high BMI across health perspectives. However, studies data that pointed to the relationship between obesity and academic performance in the capital of Saudi Arabia are few.

Obesity is defined as an abnormal or excessive accumulation of fat in the body that presents a risk to health [5]. In this study, the BMI has been used to measure and evaluate obesity among study participants. BMI pertains to a person’s weight in kilograms divided by the square of height in meters [6]. It is categorized into three classes, namely, Class 1: low-risk obesity (BMI = 30.0 to 34.9), Class 2: moderate-risk obesity (BMI = 35.0 to 39.9), and Class 3: high-risk obesity (BMI ≥ 40.0). A person is considered overweight but not obese if BMI = 25.0 to 29.9 [7].

The majority of the studies propose that a relationship exists between increased BMI in the case of obesity/overweight and a decline in academic performance. However, Alswat et al. [8] conducted a study in Taif city in Saudi Arabia and found no association between BMI and academic performance, except for academic performance in physics, where students with obesity obtained lower grades than students with average BMI [8]. Moreover, a few studies examined the same aspect from the perspective of gender and observed no difference between males and females [8-9].

Alternatively, obesity is not only associated with academic performance but also with other factors, such as paternal and maternal education level, family income, increasing number of family members, living with whom, marital state and health behaviors. Almoajel et al. [10] conducted a study on three colleges in King Saud University and found that high levels of education among parents exerted an influence on female children and increased their academic performance. Conversely, families with low income indicated a weak, positive association with low grade point average (GPA). Furthermore, an increase in the number of family members exerted a negative impact on GPA. Students living with parents exhibited high grades than those living without. In addition, marital state exerted an effect on GPA. In addition to the above-mentioned factors, a strong association has been noticed in two colleges, namely, science and medicine. In the College of Science, students with physical activity exhibit higher GPA than those without. Alternatively, in the College of Medicine, students with unhealthy sleep patterns and stress scored lower GPA than those without [10].

A healthy diet is one of the health behaviors not mentioned in a previous study on King Saud University [10]. However, Stea and Torstveit [9] conducted a study in Norway and reported the impact of diet on academic achievement. The authors found a strong association of regular breakfast and lunch and consumption of fruit and vegetables to improved academic performance for male and female students, while drinking lemonade, smoking, and snuffing displayed a negative influence on academic performance for both genders. However, an association was found between a high consumption of ready-made food, such as sugar-sweetened drinks and salty snacks and decreased academic performance only among female students. Moreover, students with physical activities in their leisure time produced high grades [9].

Although the number of studies is few, and the outcomes measured by these researchers are distinct, several remarkable strong points can be obtained. Nevertheless, when taken together, the results of these studies are relatively consistent. In summary, the majority of studies observed an association between obesity, unhealthy lifestyle, and academic performance. However, the evidence remains unclear. As such, many research gaps should be addressed to obtain clear outcomes.

Toward this end, the present study aimed to examine the relationship between obesity, unhealthy lifestyle, and academic performance of female students in the College of Science and College of Medicine of IMAMU, Riyadh, Saudi Arabia.

## Materials and methods

The researchers conducted an observational, descriptive, and cross-sectional study through a survey. The study was conducted in two phases because of the contrasting nature of the subjects. Moreover, there are subjects in medicine with high credit hours compared to lower ones in the College of Science. For the previous reason the comparison will be more effective, and more association will be determined. In addition, because of the minimal number of students in the college of medicine, the college of science was added to augment the sample size and more generalization can be determined. The two phases did not include any adding or modifications in the questionnaire, only questions on math and physics grades were added, therefore, it did not affect the analysis. The first was conducted in the College of Medicine, where we evaluated approximately 153 students. For data collection, the participants completed a questionnaire, which was administered by one of the researchers. The second phase was conducted at the College of Science using the same questionnaire with 175 students. In the second phase, however, questions on math and physics were added to the questionnaire, because evidence suggests that these subjects are mainly related to the mental ability by utilizing problem-solving skills. The total number of participants was 328 female students from both colleges. The study only involved female students for the reason that the College of Science and College of Medicine campus contains females only, while the males are centered in another campus. Any participant who had diabetes mellitus, hypertension, asthma, or other chronic diseases was excluded from the study because of its possibility to impact on the mental status, hence we wanted to include all apparently healthy individuals to avoid any other influencing factors that could cause possible bias in the study.

Sample size calculation

Based on the population size equal to 2168, which represents the number of students in both College of Science and College of Medicine, the sample size is assumed to be 327 participants based on the sample size calculation formula: SS = [Z2p (1 − p)]/ C2 with confidence interval (CI): 95%, Population Proportion of 50% and margin of error of 5.

Data collection and instrument

The questionnaire included items on routine physical activity, eating habits, GPA, family income and level of education. Weight and height scales were used for anthropometric measurements. The data was collected through a survey in which the BMI (weight in kilograms divided by the square of height in meters) was measured and calculated in the IMAMU medical services and hence recorded in the questionnaire. The survey was conducted on December 12, 2020 and June 15, 2021. The questionnaire was administered by a researcher through Google Forms electronic survey.

Statistical analysis

All categorical variables, such as college level and GPA, were presented as numbers and percentages. Continuous variables, such as age, weight, height, and BMI, were expressed as mean ± standard deviation. Independent sample t-test was used to determine the mean significant difference between obesity and the characteristics of the participants. Pearson’s Chi-square/Fisher’s exact test was applied according to whether the cell-expected frequency is less than 5 and to determine the significant relationship between categorical variables. A *p*-value of less than 0.05 was considered statistically significant. All data were entered and analyzed using SPSS 22 (IBM Corp., Armonk, USA).

## Results

After excluding students with diabetes and/or other diseases, a total of 308 students were included for analysis (n = 152 from the College of Medicine; n = 156 from the College of Science). Table 1 presents the basic information of the participants, Table 2 provides their dietary characteristics and Table 3 displays further detailed analysis.

**Table 1:** Demographic and Clinical Characteristic of the Participants ^a^GPA: Grade point average. ^b^BMI: Body mass index

	College of Medicine	College of Science	*p*-Value
	n = 152	n = 156	
Level (year)			<0.001
1–2 (1)	62 (40.8%)	62 (39.7%)	
3–4 (2)	66 (43.4%)	43 (27.6)	
5–6 (3)	24 (15.8)	27 (17.3%)	
7–8 (4)	0 (0.0%)	24 (15.4%)	
Group GPA^a^			<0.001
≤3.0	1 (0.7%)	43 (27.6%)	
>3.0 to ≤4.5	107 (70.3%)	101 (64.7%)	
>4.5	44 (28.9%)	12 (7.7%)	
BMI^b^ Classification			0.335
Underweight	13 (8.6%)	20 (12.8%)	
Normal	91 (59.9%)	79 (50.6%)	
Overweight	32 (21.1%)	41 (26.3%)	
Obese	16 (10.5%)	16 (10.3%)	
Obese and Non-Obese			0.359
Obese	48 (31.6%)	57 (36.5%)	
Non-Obese	104 (68.4%)	99 (63.5%)	
Physical activities			<0.001
Walking	85 (55.9%)	58 (37.2%)	
Cycling	5 (3.3%)	6 (3.8%)	
Rope workout	4 (2.6%)	18 (11.5%)	
All	14 (9.2%)	34 (21.8%)	
None	44 (28.9%)	40 (25.6%)	
Group Meals			<0.001
≤3	132 (86.8%)	156 (100.0%)	
>3	20 (13.2%)	0 (0.0%)	
Family income			<0.001
2000–5000	3 (2.0%)	12 (7.7%)	
5000–8000	4 (2.6%)	32 (20.5%)	
8000–11000	28 (18.4%)	44 (28.2%)	
>11000	117 (77.0%)	68 (43.6%)	
Mother's level of education			<0.001
Elementary	5 (3.3%)	35 (22.4%)	
Intermediate	8 (5.3%)	16 (10.3%)	
High School	28 (18.4%)	26 (16.7%)	
Bachelor	82 (53.9%)	49 (31.4%)	
Masters	18 (11.8%)	19 (12.2%)	
PhD	11 (7.2%)	11 (7.1%)	
Father's level of education			<0.001
Elementary	4 (2.6%)	17 (10.9%)	
Intermediate	5 (3.3%)	6 (3.8%)	
High School	23 (15.1%)	52 (33.3%)	
Bachelor	54 (35.5%)	33 (21.2%)	
Masters	25 (16.4%)	17 (10.9%)	
PhD	41 (27.0%)	31 (19.9%)	

**Table 2:** Dietary Characteristics of the Participants

	College of Medicine	College of Science	*p*-Value
	n = 152	n = 156	
Eating while watching TV/using mobile phones			<0.001
Always	78 (51.3%)	53 (34.0%)	
Sometimes	48 (31.6%)	67 (42.9%)	
Never	26 (17.1%)	36 (23.1%)	
Diet/healthy eating			<0.001
Always	50 (32.9%)	81 (51.9%)	
Sometimes	74 (48.7%)	32 (20.5%)	
Never	28 (18.4%)	43 (27.6%)	
Having breakfast			0.842
Always	58 (38.2%)	56 (35.9%)	
Sometimes	61 (40.1%)	62 (39.7%)	
Never	33 (21.7%)	38 (24.4%)	
Having dinner			0.296
Always	46 (30.3%)	57 (36.5%)	
Sometimes	70 (46.1%)	72 (46.2%)	
Never	36 (23.7%)	27 (17.3%)	
Eating fresh fruits/veggies			0.844
Always	52 (34.2%)	57 (36.5%)	
Sometimes	82 (53.9%)	79 (50.6%)	
Never	18 (11.8%)	20 (12.8%)	
Eating yogurt			0.153
Always	32 (21.1%)	41 (26.3%)	
Sometimes	84 (55.3%)	69 (44.2%)	
Never	36 (23.7%)	46 (29.5%)	
Eating potato chips			0.273
Always	38 (25.0%)	52 (33.3%)	
Sometimes	88 (57.9%)	81 (51.9%)	
Never	26 (17.1%)	23 (14.7%)	
Drink fresh juice			0.056
Always	27 (17.8%)	45 (28.8%)	
Sometimes	71 (46.7%)	68 (43.6%)	
Never	54 (35.5%)	43 (27.6%)	
Drinking ready-made juice			0.046
Always	24 (15.8%)	40 (25.6%)	
Sometimes	68 (44.7%)	71 (45.5%)	
Never	60 (39.5%)	45 (28.8%)	
Drinking milk			0.873
Always	63 (41.4%)	62 (39.7%)	
Sometimes	52 (34.2%)	52 (33.3%)	
Never	37 (24.3%)	42 (26.9%)	
Drinking water			<0.001
Always	104 (68.4%)	64 (41.0%)	
Sometimes	43 (28.3%)	92 (59.0%)	
Never	5 (3.3%)	0 (0.0%)	

**Table 3:** Descriptive Analysis of Study Characteristics ^a^GPA: Grade point average

	Minimum	Maximum	Median	Medicine	Science	Total
				Mean ± SD	Mean ± SD	Mean ± SD
Age	18	35	21	20.39 ± 1.24	21.56 ± 2.21	20.99 ± 1.89
GPA^a^	1.69	5.40	4.00	4.21 ± 0	3.43 ± 0.78	3.81 ± 0.76
Weight (Kg)	33.00	115.00	59.00	61.4 ± 12.48	60.07 ± 13.74	60.73 ± 13.13
Height (m)	1.46	1.75	1.60	1.61 ± 0.06	1.59 ± 0.06	1.6 ± 0.06
Body Mass Index	14.06	43.28	23.12	23.78 ± 4.47	23.65 ± 5.02	23.72 ± 4.75
Meals per day	1	10	2	2.57 ± 1.2	2.08 ± 0.54	2.32 ± 0.96
Sleeping hours	2	14	6	6.72 ± 2.1	7.45 ± 2.14	7.09 ± 2.15
Hours spent watching TV/on social media	1	24	6	5.6 ± 3.5	6.88 ± 2.45	6.25 ± 3.07

Table 4 and Table 5 present the association between obesity, unhealthy habits, and academic performance as well as the impact of family income and level of education of parents on the academic performance of students. Table 6 illustrates the association between obesity and grades for physics and math in the College of Science to determine if a further association between obesity and academic performance could be obtained.

**Table 4:** Association Between Obesity, Unhealthy Habits, and Academic Performance (GPA = 5) ^a^GPA: Grade point average. ^b^BMI: Body mass index.

	College of Science			College of Medicine			*p*-Value
	≤3.0	>3.0 to ≤4.5	> 4.5	≤3.0	>3.0 to ≤4.5	> 4.5	
GPA^a^ Classification	43 (27.6%)	101 (64.7%)	12 (7.7%)	1 (0.7%)	107 (70.3%)	44 (28.9%)	
BMI^b^							0.335
Obese	11 (25.6%)	43 (42.6%)	3 (25.0%)	0 (0.0%)	42 (39.3%)	6 (13.6%)	
Non-obese	32 (74.4%)	58 (57.4%)	9 (75.0%)	1 (100.0%)	65 (60.7%)	38 (86.4%)	
Physical activity							0.086
Walking	16 (37.2%)	38 (37.6%)	4 (33.3%)	0 (0.0%)	62 (57.9%)	23 (52.3%)	
Cycling	1 (2.3%)	5 (5.0%)	0 (0.0%)	0 (0.0%)	4 (3.7%)	1 (2.3%)	
Rope workout	3 (7.0%)	12 (11.9%)	3 (25.0%)	0 (0.0%)	2 (1.9%)	2 (4.5%)	
All	6 (14.0%)	23 (22.8%)	5 (41.7%)	1 (100.0%)	8 (7.5%)	5 (11.4%)	
None	17 (39.5%)	23 (22.8%)	0 (0.0%)	0 (0.0%)	31 (29.0%)	13 (29.5%)	
Dieting/healthy eating							0.309
No	16 (37.2%)	26 (25.7%)	1 (8.3%)	0 (0.0%)	18 (16.8%)	10 (22.7%)	
Maybe	9 (20.9%)	20 (19.8%)	3 (25.0%)	0 (0.0%)	54 (50.5%)	20 (45.5%)	
Yes	18 (41.9%)	55 (54.5%)	8 (66.7%)	1 (100.0%)	35 (32.7%)	14 (31.8%)	
Having breakfast							0.637
6–10 times/week	19 (44.2%)	33 (32.7%)	4 (33.3%)	1 (100.0%)	40 (37.4%)	17 (38.6%)	
1–5 times/week	13 (30.2%)	44 (43.6%)	5 (41.7%)	0 (0.0%)	44 (41.1%)	17 (38.6%)	
0 times/w	11 (25.6%)	24 (23.8%)	3 (25.0%)	0 (0.0%)	23 (21.5%)	10 (22.7%)	
Having dinner							0.572
6–10 times/week	18 (41.9%)	36 (35.6%)	3 (25.0%)	1 (100.0%)	33 (30.8%)	12 (27.3%)	
1–5 times/week	17 (39.5%)	47 (46.5%)	8 (66.7%)	0 (0.0%)	48 (44.9%)	22 (50.0%)	
0 times/w	8 (18.6%)	18 (17.8%)	1 (8.3%)	0 (0.0%)	26 (24.3%)	10 (22.7%)	
Eating fresh fruits/veggies							0.050
6–10 times/week	17 (39.5%)	34 (33.7%)	6 (50.0%)	0 (0.0%)	39 (36.4%)	13 (29.5%)	
1–5 times/week	20 (46.5%)	54 (53.5%)	5 (41.7%)	0 (0.0%)	58 (54.2%)	24 (54.5%)	
0 times/week	6 (14.0%)	13 (12.9%)	1 (8.3%)	1 (100.0%)	10 (9.3%)	7 (15.9%)	
Eating yogurt							0.048
6–10 times/week	13 (30.2%)	26 (25.7%)	2 (16.7%)	1 (100.0%)	19 (17.8%)	12 (27.3%)	
1–5 times/week	14 (32.6%)	49 (48.5%)	6 (50.0%)	0 (0.0%)	57 (53.3%)	27 (61.4%)	
0 times/week	16 (37.2%)	26 (25.7%)	4 (33.3%)	0 (0.0%)	31 (29.0%)	5 (11.4%)	
Eating potato chips							0.084
6–10 times/week	13 (30.2%)	34 (33.7%)	5 (41.7%)	0 (0.0%)	27 (25.2%)	11 (25.0%)	
1–5 times/week	21 (48.8%)	56 (55.4%)	4 (33.3%)	0 (0.0%)	66 (61.7%)	22 (50.0%)	
0 times/week	9 (20.9%)	11 (10.9%)	3 (25.0%)	1 (100.0%)	14 (13.1%)	11 (25.0%)	
Drinking ready-made juice							
6–10 times/week	9 (20.9%)	25 (24.8%)	6 (50.0%)	0 (0.0%)	17 (15.9%)	7 (15.9%)	0.266
1–5 times/week	20 (46.5%)	46 (45.5%)	5 (41.7%)	0 (0.0%)	48 (44.9%)	20 (45.5%)	
0 times/week	14 (32.6%)	30 (29.7%)	1 (8.3%)	1 (100.0%)	42 (39.3%)	17 (38.6%)	
Drinking milk							0.518
6–10 times/week	15 (34.9%)	42 (41.6%)	5 (41.7%)	1 (100.0%)	46 (43.0%)	16 (36.4%)	
1–5 times/week	13 (30.2%)	36 (35.6%)	3 (25.0%)	0 (0.0%)	38 (35.5%)	14 (31.8%)	
0 times/week	15 (34.9%)	23 (22.8%)	4 (33.3%)	0 (0.0%)	23 (21.5%)	14 (31.8%)	
Drinking water							0.880
<1 L/day	17 (39.5%)	41 (40.6%)	6 (50.0%)	1 (100.0%)	75 (70.1%)	28 (63.6%)	
1–1.8 L/day	26 (60.5%)	60 (59.4%)	6 (50.0%)	0 (0.0%)	29 (27.1%)	14 (31.8%)	
>1.8 L/day	0 (0.0%)	0 (0.0%)	0 (0.0%)	0 (0.0%)	3 (2.8%)	2 (4.5%)	
Duration of sleep (hour)							0.142
<4	0 (0.0%)	0 (0.0%)	0 (0.0%)	0 (0.0%)	4 (3.7%)	0 (0.0%)	
4–6	16 (37.2%)	58 (57.4%)	7 (58.3%)	1 (100.0%)	50 (46.7%)	27 (61.4%)	
7–9	19 (44.2%)	27 (26.7%)	2 (16.7%)	0 (0.0%)	40 (37.4%)	15 (34.1%)	
10–12	8 (18.6%)	16 (15.8%)	3 (25.0%)	0 (0.0%)	11 (10.3%)	2 (4.5%)	
>12	0 (0.0%)	0 (0.0%)	0 (0.0%)	0 (0.0%)	2 (1.9%)	0 (0.0%)	
Hours spent watching TV/social media							0.049
<4	0 (0.0%)	0 (0.0%)	0 (0.0%)	0 (0.0%)	27 (25.2%)	19 (43.2%)	
4–6	24 (55.8%)	66 (65.3%)	8 (66.7%)	0 (0.0%)	42 (39.3%)	14 (31.8%)	
7–9	14 (32.6%)	20 (19.8%)	0 (0.0%)	0 (0.0%)	21 (19.6%)	6 (13.6%)	
10–12	5 (11.6%)	15 (14.9%)	4 (33.3%)	1 (100.0%)	16 (15.0%)	4 (9.1%)	
>12	0 (0.0%)	0 (0.0%)	0 (0.0%)	0 (0.0%)	1 (0.9%)	1 (2.3%)	

**Table 5:** Impact of Family Income and Level of Education of Parents on Academic Performance of the Participants (GPA = 5) GPA: Grade point average

	College of Science			College of Medicine			*p*-Value
	≤3.0	>3.0 to ≤4.5	>4.5	≤3.0	>3.0 to ≤4.5	>4.5	
	n = 43	n = 101	n = 12	n = 1	n = 107	n = 44	
Family income							0.162
2000–5000	2 (4.7%)	9 (8.9%)	1 (8.3%)	0 (0.0%)	0 (0.0%)	3 (6.8%)	
5000–8000	5 (11.6%)	23 (22.8%)	4 (33.3%)	0 (0.0%)	2 (1.9%)	2 (4.5%)	
8000–11000	15 (34.9%)	27 (26.7%)	2 (16.7%)	0 (0.0%)	19 (17.8%)	9 (20.5%)	
>11000	21 (48.8%)	42 (41.6%)	5 (41.7%)	1 (100.0%)	86 (80.4%)	30 (68.2%)	
Mother's level of education							0.310
Elementary	7 (16.3%)	27 (26.7%)	1 (8.3%)	0 (0.0%)	3 (2.8%)	2 (4.5%)	
Intermediate	6 (14.0%)	7 (6.9%)	3 (25.0%)	0 (0.0%)	6 (5.6%)	2 (4.5%)	
High School	10 (23.3%)	14 (13.9%)	2 (16.7%)	0 (0.0%)	19 (17.8%)	9 (20.5%)	
Bachelor	12 (27.9%)	34 (33.7%)	3 (25.0%)	1 (100.0%)	57 (53.3%)	24 (54.5%)	
Masters	5 (11.6%)	11 (10.9%)	3 (25.0%)	0 (0.0%)	14 (13.1%)	4 (9.1%)	
PhD	3 (7.0%)	8 (7.9%)	0 (0.0%)	0 (0.0%)	8 (7.5%)	3 (6.8%)	
Father's level of education							0.504
Elementary	4 (9.3%)	13 (12.9%)	0 (0.0%)	0 (0.0%)	3 (2.8%)	1 (2.3%)	
Intermediate	1 (2.3%)	5 (5.0%)	0 (0.0%)	0 (0.0%)	4 (3.7%)	1 (2.3%)	
High School	16 (37.2%)	31 (30.7%)	5 (41.7%)	0 (0.0%)	18 (16.8%)	5 (11.4%)	
Bachelor	12 (27.9%)	20 (19.8%)	1 (8.3%)	0 (0.0%)	36 (33.6%)	18 (40.9%)	
Masters	4 (9.3%)	12 (11.9%)	1 (8.3%)	1 (100.0%)	18 (16.8%)	6 (13.6%)	
PhD	6 (14.0%)	20 (19.8%)	5 (41.7%)	0 (0.0%)	28 (26.2%)	13 (29.5%)	

**Table 6:** Association Between Obesity and Grades in Physics and Math (College of Science) ^a^BMI: Body mass index

	≤3.0	>3.0 to ≤4.5	>4.5	*p*-Value
	(C/D+/D/F)	(B+/B/C+)	(A+/A)	
Physics grade	59 (37.8%)	63 (40.4%)	34 (21.8%)	
BMI^a^				0.893
Obese	29 (49.2%)	20 (31.7%)	10 (29.4%)	
Non-obese	30 (50.8%)	43 (68.3%)	24 (70.6%)	
Math grade	74 (47.4%)	50 (32.0%)	32 (20.5%)	
BMI^a^				0.872
Obese	28 (37.8%)	21 (42.0%)	10 (31.3%)	
Non-obese	46 (62.2%)	29 (58.0%)	22 (68.8%)	

The participants from each college were classified into three groups according to GPA. For the College of Medicine, we found that 0.7%, 70.3%, and 28.9% of students have GPAs of 3.0 or lower, 3.0-4.5, and 3.67 or higher, respectively; where GPA of 5 is the highest. In the College of Science, we found that 27.6%, 64.7%, and 7.7% of students have GPAs of 3.0 or lower, 3.0-4.5, and 3.67 or higher, respectively; where GPA of 5 is the highest (Tables 4-6).

Academic performance and health, sociodemographic dimensions

Impact of a Healthy Diet and Physical Activity on Academic Performance

Table 4 displays data from the questionnaires and indicates no association between academic performance and health. In other words, healthy eating does not seem to be related with academic performance for students from colleges (*p* = 0.309). Moreover, no association was found between academic performance and having breakfast (*p* = 0.637), having dinner (*p* = 0.572), drinking ready-made juice (*p* = 0.266), drinking milk (p = 0.518), and drinking water (*p* = 0.880). A weak association was found between physical activity (*p* = 0.086), eating fresh fruits and veggies (*p* = 0.050), and eating potato chips (0.084). Nevertheless, eating yogurt at least 1-5 times per week indicated a strong association with high GPA for both colleges (*p* < 0.05).

Association Between Obesity and Academic Performance

For both colleges, no association was found between GPA and BMI. In other words, obesity in both groups does not lead to low GPA. Thus, the null hypothesis is supported (*p *= 0.335). However, for further specificity, we examined the grades in math and physics of students from the College of Science (Table 6) to evaluate their problem-solving skills, which is one of the most important mental skills. We found no association between grades in math and physics with GPA. As predicted, the percentages demonstrate that non-obese students obtained higher grades in physics. Thus, the null hypothesis is supported (grades in physics: *p* = 0.893; math: *p* = 0.872).

The Impact of Sleep Pattern and Hours Spent Watching TV or Using Social Media on Academic Performance

The study found no association between sleep pattern and academic performance for both groups of students. Thus, the null hypothesis is supported (*p* = 0.142). However, the association between hours spent watching TV or using social media and GPA was significant for both groups (*p* < 0.05; Table 4).

Impact of Family Income and Level of Education on Academic Performance

No association was observed between family income and academic performance (*p* = 0.142). Moreover, neither the mother’s nor father’s level of education exerted an impact on academic performance (*p* = 0.310 and *p* = 0.504, respectively, Table 5). Thus, the null hypothesis is supported for both cases.

## Discussion

This study examined GPA among female students in the College of Science and College of Medicine of IMAMU in comparison to BMI, unhealthy habits, and other sociodemographic variables to establish an association among them.

Obesity does not influence academic performance for students from both colleges. The study further investigated the grades for math and physics among students from the College of Science. The results indicated that the high number of non-obese students at the College of Science exhibited better academic performance in physics. In contrast, students with obesity at the same college produced low grades in physic and middle and low grades in math. This result is in line with those of previous studies, that is, no association was established between academic performance and obesity [8, 11]. Based on a rigorous and comprehensive systematic review done by Santana et al. [12] which included qualitative analyses of the studies, it’s concluded that obesity is not statistically associated with poor academic performance in school-aged children. However, in Taras study [13], it was discovered that overweight and obesity are associated with lower academic performance.

Sociodemographic variables include income and levels of education of mothers and fathers. The average family income of medical students was higher than those of students from the College of Science. Although GPA among the majority of the respondents was within >3 and <4.5, no association was identified between low income and academic performance (*p* = 0.162). Moreover, the level of education of parents was classified into undergraduate degrees, bachelor’s degree, and postgraduate degree. Mothers with undergraduate degrees obtained a GPA of 3 and <4.5 for both colleges, whereas a small percentage of students from the College of Science obtained a GPA of ≤3. In the College of Medicine, no students with mothers with undergraduates reached a GPA of ≤3. Further investigation revealed that bachelor’s degrees were the most predominant of all degrees. Students from the College of Medicine with mothers who are holders of bachelor’s degrees are dispersed between GPA >3 and <4.5, whereas none reached a GPA of ≤3. Mothers of students from the College of Science with a bachelor’s degree achieved a GPA of ≤3, whereas a high percentage of GPA ranged between >3 and <4.5. Among mothers of students from both colleges with post-graduate degrees, a small group of students indicated a GPA of ≤3, whereas many students from both groups have a GPA of >3 and <4.5. Thus, no association was established between the level of education of mothers and GPA (*p* = 0.310). In terms of the level of education of fathers, female students whose fathers achieved undergraduate degrees accomplished GPA ≤3, which is a minor distribution. However, a slightly larger distribution was noted for GPA between >3 and <4.5. The results do not support the hypothesis that high GPA is achieved among students from the College of Science. For students from the College of Medicine, no low GPA was recorded among those whose fathers achieved undergraduate degrees and was mainly distributed between >3 and <4.5. Students from the College of Medicine whose fathers completed bachelor's degrees obtained high GPA, which was distributed between >3 and <4.5, whereas the GPA of students from the College of Science reached ≤3 with a slightly higher GPA of >3 and <4.5. The fathers of students from both colleges with post-graduate degrees achieved high GPA >3 and <4.5. The lack of association suggests that the fathers’ level of education did not influence academic performance (*p* = 0.504). However, this result does not support the finding of other studies on the effect of the levels of education of parents and family income on academic performance [14].

Undoubtedly, healthy or unhealthy lifestyle habits are interrelated. Florence et al. [11] conducted a study and found students that exhibited healthy patterns by eating healthy and regularly exercising typically displayed good physical health and outstanding academic performance [11]. Based on the systemic review of five studies, positive associations between diet and academic achievement were found, including breakfast, regular meal consumption, and meeting national recommendations for fruit intake [15]. Moreover, our study has found a significant association related to eating yogurt at least 1-5 times per week and achieving high academic performance (*p* < 0.05). Several studies support the positive impact of physical activity on academic performance [16-18]. Throughout the current study, the respondents displayed a weak association (*p* = 0.086) between physical activity and GPA performance. This result is in agreement with those of previous studies that rejected the correlation. Social media is another influencing factor in this study. The majority of respondents used social networking for approximately 4-6 h/day or as much as 7-9 h/day. The findings support the strong association between GPA and social networking (*p* < 0.05).

This study suggests that academic performance is not influenced by sleeping patterns. The majority of the respondents spent an average of 4-6 h/day and 7-9 h/day on sleeping. During the investigation, no association was found between academic performance and sleep patterns. However, this result does not support the finding of other studies, which stated that insomnia and other sleeping disorders are related to low GPA [19, 20].

In summary, the present study found that obesity does not influence academic performance among students from both colleges in IMAMU. Moreover, the results do not provide support for the association among the level of education of parents, family income, and sleeping hours on the academic performance of the female students. High levels of education among parents may increase the probability of high GPA. However, other studies have disproved this theory [21]. Moreover, physical activity and eating yogurt exerted a positive impact on academic performance or led to high GPA.

Limitations of the study

This study has its limitations. The first is that the sample size is small, which may influence the accuracy of the results. Second, similar to any cross-sectional study, the reported values may be a snapshot and not represent the full experience of this population. In addition, the study was unable to address the effects of lifestyle patterns, which vary among individuals. Moreover, the prevalence of obesity may exhibit variations that may have influenced the outcome. The study was a single-center study, such that the observations of the present study may be inapplicable to the general population in the Kingdom of Saudi Arabia. In addition, the study was conducted in female colleges. Thus, the study was unable to examine differences in terms of gender.

## Conclusions

Academic performance is influenced by many factors. Eating yogurt more frequently and watching TV and spending less time on social media are the most influential ones on academic performance. However, obesity is not a factor that exerts an influence on academic performance. Moreover, the study found no direct effect of physical activity and healthy diet on academic performance.

Large-scale multicenter studies are recommended to validate the findings of the present study. Furthermore, college and university professors, staff, and administrators must be aware of the results, because they indicate that health promotion can improve body weight and total body fat percentage, which may enhance academic performance. Measurements of lean muscle mass, total body fat mass, hip circumference, and abdominal fat are also recommended, because these factors may be more useful in examining the link among obesity, nutrition, lifestyle habits, and academic performance.
